# Supplementary material for: TRI-POSE-Net: Adaptive 3D human pose estimation through selective kernel networks and self-supervision with trifocal tensors
Source: PLoS One. 2024 Dec 5;19(12):e0310831. doi: 10.1371/journal.pone.0310831 (PMC11620359; doi:10.1371/journal.pone.0310831)
Supplement: Attachment — Submitted filename: R1_Tri_Pose_Response_Letter.pdf [file pone.0310831.s001.pdf]

Manuscript ID: PONE-D-24-10615

Article title: TRI-POSE-Net: Adaptive 3D Pose Estimation in the Metaverse through Selective Kernel Networks and Self-Supervision with Trifocal Tensors

Plos One

May 5, 2024

Dear Editor-in-Chief,

First of all, we would like to extend our sincere gratitude to the Editor, for allowing a re-submission of our manuscript, with an opportunity to address the reviewers' comments. We greatly appreciate the reviewers' time spent reviewing the paper and suggesting valuable suggestions to improve the quality of the paper. We have studied the comments carefully and made necessary corrections as advised by the worthy reviewers. We have revised the paper to reflect all the comments made by the Associate Editor and anonymous reviewers. These changes are fully addressed in this reply letter.

Below, we provide point-to-point responses explaining how we addressed each of the Reviewer's comments. For clarity, our responses have been written in blue color. When we copy the text from the manuscript, it is given in red color.

Reviewer 1

Reviewer # 1 (Concern # 1)

The two aspects of the contribution (architecture and self-supervision) have little to do with each other, and neither are properly evaluated on their own. If the central point is the architecture, the choices should be better justified and evaluated, for example looking at the impact of SKNet blocks on different backbones, and looking at the results on different datasets. If the self-supervision is the central aspect, its benefits should be verified on different models, and more in-depth analysis of the quality of the generated groundtruth would be beneficial. It would also be interesting to look at whether that supervision improves upon the 2d joint position prediction once reprojected, or if the results are only as good as (or worse than) the 2d joint position estimation network used to create the annotation.

Response: The authors are grateful for your valuable suggestions. We reflected your suggestions in the related work and results section.

Reviewer # 1 (Concern # 2)

The method presented also doesn't outperform comparable state-of-the-art (3 reported methods outperform it, despite it using an additional dataset as pre-training)

Response: Respected reviewer, we have updated table 3 and 4 to address this concern that how our proposed model is superior than the others.

Reviewer # 1 (Concern # 3)
The reference presented do not always include all of the relevant informations, especially the conference venues. Many of them also refer to Github repository. No reference to the paper introducing ResNet or SKNet is done in the introduction despite discussing it.

Response: The authors appreciate your concerns. We have updated our references and removed conference venues references that are less important.

Reviewer # 1 (Concern # 4)
While the problem of fast and accurate Human Pose Estimation is relevant and of interest to this venue, nothing in the method presented relate it specifically to the "metaverse" (which is not a well-defined technical term in the first place), so putting it in the title seems unnecessary.

Response: Thank you very much. Metaverse domain is added for the special issue in which we tried our paper before. Now we have removed all terms related to metaverse and updated the paper.

Reviewer # 1 (Concern # 5)
the title should state Human Pose Estimation instead of Pose Estimation

Response: Respected reviewer, we have updated the title as "TRI-POSE-Net: Adaptive 3D Human Pose Estimation Through Selective Kernel Networks and Self-Supervision with Trifocal Tensors"

Reviewer # 1 (Concern # 6)
restraints instead of constraints

Response: Respected reviewer, we have corrected this mistake.

Reviewer # 1 (Concern # 7)
enlisted instead of listed

Response: Respected reviewer, we have corrected this mistake.

Reviewer # 1 (Concern # 8)
parametric sharing instead of parameter sharing

Response: Respected reviewer, we have corrected this mistake.

Reviewer # 1 (Concern # 9)
lineament is not clear, possibly misused

Response: Respected reviewer, we have corrected this mistake.

Reviewer # 1 (Concern # 10)
N and A used in 3.1 before being defined

Response: Respected reviewer, we have added details of N and A in the paper.

Reviewer # 1 (Concern # 11)
Aec and Bec used without being defined in equation 5

Response: Respected reviewer, we have added details of Aec and Bec in the paper.

Reviewer # 1 (Concern # 12)
the text of Figure 3 and 7 is a little too small

Response: Respected reviewer, we have improved the quality of the figure to make it readable.

Reviewer # 1 (Concern # 13)
In figure 8, the keypoints are not visible

Response: Respected reviewer, we have improved the quality of the figure 8 to make it readable.

Reviewer 2

Reviewer # 2 (Concern # 1)

Why the authors forcefully adding the terms of meta-verse in the paper? I would suggest if author remove the terms of metaverse from the paper.

Response: We are thankful for your feedback and appreciate your concern. Respected Reviewer, we have removed the term metaverse completely from the paper.

Reviewer # 2 (Concern # 2)

Add the results of the proposed model in the abstract.

Response: Thank you very much for your valuable comment. Respected reviewer, we have added the results in the abstract section.

Reviewer # 2 (Concern # 3)

Add some potential references in the paper replacing too much conference and website links from the paper.

Response: We appreciate your insightful suggestion. Respected reviewer, we have updated the conference articles with research articles to improve this part.

Reviewer # 2 (Concern # 4)

Add some details (single paragraph) about related work section before moving to section 2.1 directly.

Response: Thank you very much for this suggestion. we have added a paragraph to describe the related work section.

Reviewer # 2 (Concern # 5)

In section 2.2, references 11-13 and 14-17 are not cited properly, try to discuss them separately or remove less relevant papers.

Response: Respected reviewer, we have corrected these references

Reviewer # 2 (Concern # 6)

There are too many short forms used in the paper. Try to add abbreviation table to improve the readability of the paper.

Response: Respected reviewer, we have added acronym table at the end of conclusion section.

Reviewer # 2 (Concern # 7)

There is no graph to visualize the results. Remembering values is difficult.

Response: Thank you very much for pointing out this lack. We have added figure 9 to address this concern.

Reviewer # 2 (Concern # 8)

Explain how MPJPE is best evaluation parameter for this model.

Response: Respected reviewer, we have added these lines to address this comment:

The Mean Per Joint Position Error (MPJPE) stands out as a preferred evaluation parameter for human 3D pose estimation due to its ability to directly measure the accuracy of estimated poses by calculating the Euclidean distance between corresponding joints in predicted and ground truth poses [**?**]. This joint-level evaluation offers a detailed assessment of algorithm performance across different body parts, enabling researchers to identify specific areas for improvement. Moreover, MPJPE's robustness to pose variability and its ease of interpretation make it widely adopted as a standard metric in the field, ensuring consistent and reproducible evaluation across various datasets and algorithms. Its comprehensive nature, combined with its widespread adoption, solidifies MPJPE's position as a reliable and effective evaluation metric for human 3D pose estimation.

Reviewer # 2 (Concern # 9)

Re-write conclusion section by adding result values.

Response: The authors appreciate your valuable suggestions. we have updated the conclusion section by adding result values.

Reviewer # 2 (Concern # 10)

What are the limitations of the proposed model?

Response: The authors appreciate your valuable suggestions. We have added the following limitation section the paper:

0.1 Limitation of the proposed model

The proposed model offers several advancements in the field of 3D human pose estimation. However, it also comes with certain limitations. Firstly, while the selective kernel networks and self-supervision with trifocal tensors contribute to improved performance, they also increase the complexity of the model, resulting in higher computational requirements and longer training times. Additionally, the effectiveness of the model heavily depend on the availability and quality of training data, as well as the chosen hyperparameters, which could limit its generalizability to diverse datasets and real-world scenarios. Furthermore, the proposed model may struggle with accurately estimating poses in cases where there are occlusions, ambiguous joint configurations, or extreme variations in pose appearance, which are common challenges in real-world applications. Finally, the implementation and deployment of the model may require substantial

expertise in computer vision and deep learning, making it less accessible to researchers and practitioners without specialized knowledge. These limitations highlight areas for future research and improvement to enhance the robustness, efficiency, and usability of the proposed model in practical applications.

Reviewer # 2 (Concern # 11)

English editing is necessary, there are many typos and grammatical mistake in the paper. Authors are advised to revise the paper critically.

Response: The authors appreciate your valuable suggestions. We have improved the sentence structure and correct the grammatical error throughout the paper.
