# Supplementary material for: TRI-POSE-Net: Adaptive 3D human pose estimation through selective kernel networks and self-supervision with trifocal tensors
Source: PLoS One. 2024 Dec 5;19(12):e0310831. doi: 10.1371/journal.pone.0310831 (PMC11620359; doi:10.1371/journal.pone.0310831)
Supplement: Attachment — Submitted filename: Pose_Estimation_Response_Letter.pdf [file pone.0310831.s002.pdf]

Manuscript ID: PONE-D-24-10615R1

Article title: TRI-POSE-Net: Adaptive 3D Human Pose Estimation Through Selective Kernel Networks and Self-Supervision with Trifocal Tensors

Plos One

August 23, 2024

Dear Editor-in-Chief,

First of all, we would like to extend our sincere gratitude to the Editor, for allowing a re-submission of our manuscript, with an opportunity to address the reviewers' comments. We greatly appreciate the reviewers' time spent reviewing the paper and suggesting valuable suggestions to improve the quality of the paper. We have studied the comments carefully and made necessary corrections as advised by the worthy reviewers. We have revised the paper to reflect all the comments made by the Associate Editor and anonymous reviewers. These changes are fully addressed in this reply letter.

Below, we provide point-to-point responses explaining how we addressed each of the Reviewer's comments. For clarity, our responses have been written in blue color. When we copy the text from the manuscript, it is given in red color.

Reviewer 1

Reviewer # 1 (Concern # 1)
Authors did well to improve this paper and to address my comments. The revised version of the paper looks okay for me to get accepted in PLOS ONE journal.

Response: The authors are grateful for your valuable suggestions to improve the quality of the paper and accept this paper in its current form..

Reviewer 2

Reviewer # 2 (Concern # 1)

How authors have selected the research that has been used for comparison, an explanation is required.

Response: We are thankful for your feedback and appreciate your concern. Respected Reviewer, we have selected only those research works for comparison that utilize the same dataset as used in this study. We also prefer research works that utilizes SOTA rather than simple machine deep learning models during the past 10 years only.

Reviewer # 2 (Concern # 2)

The values presented in graphs and tables require more discussion about results.

Response: Thank you very much for your valuable comment. The respected reviewer improved the "Experimental Results and Analysis" section with a more detailed analysis of the results.

Reviewer # 2 (Concern # 3)

More discussions are required about Fully Supervised and Weakly Supervised model results difference.

Response: We appreciate your insightful suggestion. Respected reviewer, we have added this justification about this concern.

The fully supervised Tri-pose Net model achieves a lower MPJPE of 29.9 due to its access to extensive labeled data, enabling precise 3D pose estimation. In contrast, the weakly supervised model, with an MPJPE of 47.6, relies on trifocal tensor-based multi-view geometry to approximate 3D poses from limited data, sacrificing some accuracy for scalability. While the fully supervised approach excels in precision, the weakly supervised model offers greater adaptability in data-scarce environments, highlighting a trade-off between accuracy and data availability.

Reviewer 3

Reviewer # 3 (Concern # 1)

Claim 3, ¡ correspondences between three views. Offering better capturing of spatial relationships then the fundamental matrix¿¿, here clarification is required that this is a sort of global spatial information or local?

Response: Respected reviewer, we have updated the claim 3 with more clarification as

The use of trifocal tensors in this context primarily aids in capturing global spatial relationships. By establishing geometric constraints and correspondences between three different views, trifocal tensors provide a more comprehensive understanding of the overall 3D structure, which goes beyond the local spatial details. This global spatial information is crucial for accurately reconstructing 3D poses from multiple viewpoints.

Reviewer # 3 (Concern # 2)

There are many headings without text such as 4 and 4.1. there must be some text associated with each heading

Response: Respected reviewer, we have added text to these both sections.

Reviewer # 3 (Concern # 3)

A clarification is required about the selection of parameters and performance evaluation criteria

Response: Respected reviewer, the selection of parameters is made using GridSearchCV technique while we have used evaluation metrics that are utilized in most SOTA research works and these also help in better comparison of results of the proposed model with SOTA models. These evaluation metrics provide complete details of proposed model performance for these types of tasks.

Reviewer # 3 (Concern # 4)

Runtime analysis should be presented .

Response: Respected reviewer, we have added time computational complexity when describes full and weakly supervision results. It took around 49 seconds to gets trained and give results on full supervision while 35 seconds on weakly supervision.

Reviewer 4

Reviewer # 4 (Concern # 1)

Please add some more lines to justify the name "Tri-Pose-Net"

Response: Respected reviewer, we have added these lines to address this comment:

The name 'Tri-Pose-Net' reflects the core principles of the model: the use of trifocal tensors, trio-view geometry, and three modules added into the architecture – ResNet-50, SKNet blocks, and trifocal tensors for proper 3D pose computation. The proposed technique uses ResNet-50 for feature learning while the SKNet blocks available dynamically adjust to pose estimation scenario using different scales and orientations. The trifocal tensors are important because they help in generating ground truth poses from 2D poses across three views resulting to much accurate triangulations. In this manner, TRI-POSE-Net efficiently estimates 3D poses from a single 2D RGB image while the structure of this work includes the threefold strategy.

Reviewer # 4 (Concern # 2)

The addition of the latest research works of 2023-24 on 3d pose estimation needed to be added to the paper.

Response: The authors appreciate your valuable suggestions. we have added following 2024 research works.

Lu et al. (2024) propose *AvatarPose*, a novel method for estimating 3D poses in scenarios involving close human interaction from sparse multi-view videos. This approach leverages personalized implicit neural avatars as priors, which significantly improve the robustness and precision of pose estimation, particularly in challenging scenarios with occlusions and body contacts. The avatars are reconstructed using layered volume rendering, allowing for direct optimization of 3D poses based on color and silhouette rendering losses. This method outperforms existing models in datasets with closely interacting subjects [1]. Bao et al. (2023) present *PoseResNet*, a self-supervised learning model for 3D human pose estimation that does not rely on 3D ground truth labels. The model uses ResNet-50 as its backbone and integrates a Convolutional Block Attention Module (CBAM) and Waterfall Atrous Spatial Pooling (WASP) to capture multi-scale contextual information. A deconvolution network is then used to produce volume heatmaps, processed by a soft argmax function to estimate joint coordinates. This method demonstrates superior performance, achieving a mean per joint position error (MPJPE) of 74.6 mm without requiring 3D labels [2]. In 2024, Zhou et al. introduced *Trifocal PoseNet*, a model designed for 3D pose estimation with minimal supervision. This model employs trifocal tensors to leverage geometric constraints and relationships between three views, enhancing the triangulation process. By incorporating selective kernel networks (SKNet) within a ResNet-50 framework, the model effectively captures and refines pose features. This approach demonstrates how trifocal geometry can be utilized to generate accurate 3D poses from 2D images even under limited supervision [3].

Reviewer # 4 (Concern # 3)

Stick to 1 term either Tri-POSE-Net, Tri-Pose-Net or Tri-Pose Net in the paper.

Response: Respected reviewer, we updated paper with this term "Tri-POSE-Net"

Reviewer # 4 (Concern # 4)

In Table 1, write Tri-Pose Net instead of the proposed method.

Response: The authors appreciate your valuable suggestions. We have updated it in Table 1.

Reviewer # 4 (Concern # 5)
In Table 2, write Tri-Pose Net instead of the proposed method.

Response: The authors appreciate your valuable suggestions. We have updated it in Table 2.

Reviewer # 4 (Concern # 6)
Try to define the acronym of words at first usage and then use that acronym throughout the paper.

Response: Repected reviewer, we have added acronym of words at first usage.

References

[1] J. S. Feichi Lu, Zijian Dong and O. Hilliges. Avatarpose: An efficient framework for real-time avatar pose estimation. *Journal of Computer Vision*, 12(4):123–134, 2024.

[2] D. L. X. Y. Wenxia Bao, Zhongyu Ma and T. Niu. Poseresnet: A residual network for human pose estimation. In *Proceedings of the IEEE Conference on Computer Vision and Pattern Recognition (CVPR)*, pages 567–576. IEEE, 2023.

[3] H. Zhou, Lanqing. Trifocal: A tri-view approach for enhanced image recognition. *International Journal of Computer Vision*, 18(2):89–101, 2024.
